# An Efficient Coding Technique for Stochastic Processes

**DOI:** 10.3390/e24010065

**Published:** 2021-12-30

**Authors:** Jesús E. Garca, Verónica A. González-López, Gustavo H. Tasca, Karina Y. Yaginuma

**Affiliations:** 1 Department of Statistics, University of Campinas, Rua Sérgio Buarque de Holanda, 651, Campinas, São Paulo 13083-859, Brazil; veronica@ime.unicamp.br (V.A.G.-L.); tasca_gustavo@hotmail.com (G.H.T.); 2 Department of Statistics, Fluminense Federal University, Rua Professor Marcos Waldemar de Freitas Reis, s/n, Niterói, Rio de Janeiro 24210-201, Brazil; karinayuriko@id.uff.br

**Keywords:** partition Markov models, Bayesian Information Criterion, Huffman coding, entropy

## Abstract

In the framework of coding theory, under the assumption of a Markov process $\left(X_{t}\right)$ on a finite alphabet A, the compressed representation of the data will be composed of a description of the model used to code the data and the encoded data. Given the model, the Huffman’s algorithm is optimal for the number of bits needed to encode the data. On the other hand, modeling $\left(X_{t}\right)$ through a Partition Markov Model (PMM) promotes a reduction in the number of transition probabilities needed to define the model. This paper shows how the use of Huffman code with a PMM reduces the number of bits needed in this process. We prove the estimation of a PMM allows for estimating the entropy of $\left(X_{t}\right)$, providing an estimator of the minimum expected codeword length per symbol. We show the efficiency of the new methodology on a simulation study and, through a real problem of compression of DNA sequences of SARS-CoV-2, obtaining in the real data at least a reduction of 10.4%.

## 1. Introduction

The research and development of data compression methods based on stochastic processes consider the frequencies of the occurrences of the elements of the underlying alphabet to produce a code. Data compression methods are designed to transmit and store data, see [1]. The Huffman coding method is an icon in this field since it is optimal to compress genetic data, text files, etc. Due to the speed with which knowledge about DNA-related structures evolves, the volume of information has grown dramatically, making it impossible to cover all the information on relevant phenomena without a strategy that allows compressing a large number of sequences currently available in databases, such as the free source, The National Center for Biotechnology Information Advances Science and Health (NCBI, accessed on 24 December 2021, https://www.ncbi.nlm.nih.gov/).

The data compression process built by Huffman coding incorporates the use of probabilities to produce an optimal code. This article seeks to improve the Huffman code’s performance about (i) how many probabilities are needed and (ii) how the probabilities are estimated. Obtaining an efficient compression method requires a deep understanding of the behavior of the database. Then, the representation that promotes compression must be generated from an appropriate stochastic model that accurately represents the database. Such notions run in parallel since they appeal to the minimum information necessary to retrieve a message or to the minimum description of a model necessary to design the behavior of a database. While in compression’s field the goal is to use a reduced number of bits to encode the message, in modeling, the goal is to identify an accurate model for the representation of the message. Then, we can think about the premise of identifying an optimal method for compression that uses an optimal stochastic model to produce the data compression. A series of aspects arises when classifying a stochastic model as optimal, plausibility, complexity, etc. In this paper, we consider these aspects and we treat them from the perspective of model selection and data compression. This problem is also related to model selection’s Minimum Description Length principle (see [2]). This principle established that the best model for a given dataset is the one that produces the shortest encoding when considering the encoding of the model plus the encoding of the dataset using the model.

A message, a DNA sequence, among other data structures, can be seen as a realization of a stochastic process, subject to a certain alphabet and with a certain memory. For example, in [3,4], it is shown that the genetic structures of SARS-CoV-2, in *FASTA* format, can be modeled as a Markovian process. In this case, a model of partitions with interstice was used, which is a variant of the model initially proposed in [5] (and investigated in [6]). In those cases, the alphabet is composed of bases, A={a,c,g,t}, where the bases are adenine (*a*), thymine (*t*), guanine (*g*), and cytosine (*c*). The dependence structure that exists in genetic sequences receives a description that is given by (i) a representation of the state space, by identifying genetic units, these units are groups of states that compound a partition of the whole state space and by (ii) the set of transition probabilities from each genetic unit to each base of A.

Throughout this paper, we are assuming that the true model generating the message is unknown and that it will be represented by a Markov chain. According to the Minimum Description Length’s principle, it is necessary to identify a parsimonious model to represent the Markov chain. The goal of this paper is to obtain an improvement in the efficiency of Huffman coding for Markov chains. Huffman’s algorithm needs to build a code for each transition probability function of the Markov process. The Partition Markov models ([6]) reduce the total number of transition probabilities needed to describe the Markov process. Then, using the partition Markov models together with the Huffman code produces a more efficient description of the model. The motivation is that we can consistently estimate the Partition Markov model to efficiently describe the message generation mechanism. We are also looking for an estimate of the minimum expected codeword length per symbol, which will allow us to validate the combined use of Huffman and partition Markov models as a cost-effective strategy.

The content of this article is as follows: Section 2 introduces the concepts and classification of coding systems. Section 3 is destined to give the main notions and way of consistent estimation of the stochastic strategy that we want to show as an option to improve the performance of the Huffman coding process. In addition, in this section, we show results establishing the relation between an optimal coding system and the estimation of a stochastic process. Section 4 exposes the stages of a message transmission process under the new strategy introduced in the previous section. Section 5 shows the advantages and disadvantages of the new strategy, compared with the application of the traditional Huffman code, and concludes with the application of the compression system to three SARS-CoV-2 sequences. The conclusions and details of the contributions of this paper follow in Section 6.

## 2. Theoretical Background and Optimal Codes

Among the qualities expected of a coding method, we note that an economy in the length of the coded message and an instantaneous method for decoding are desired, such that, it will not be necessary to receive the entire message to start the decoding process. Another aspect of coding is the complexity of the elements used for such a message since, if the complexity is high, the procedure can require high computational and memory conditions. The coding theory, see [7], organizes the problem by giving specific attributes to the encoding function, which are introduced below, associated with a random variable, and then easily extended for stochastic processes.

**Definition** **1.** Let X be a random variable taking values in A,$D^{*}$ the set of finite concatenations of elements of {0,1,2,…,D−1}. The source code of X, denoted by C, is a function $C:A\to D^{*}$ such that, for each x∈A,C(x) is the codeword of x. In addition, l(x) denotes de length of the codeword C(x).

**Definition** **2.** Let $\left(X_{1},\cdots ,X_{b}\right)$ be a size b random vector taking values in $A^{b}.$ A block source code of $(X_{1},\cdots ,X_{b}),$ (with block size b) denoted by $C^{b},$ is a function $C^{b}:A^{b}\to D^{*},$ such that, for each $\left(x_{1},\cdots ,x_{b}\right)\in A^{b},$$C^{b}\left(x_{1},\cdots ,x_{b}\right)$ is the codeword of $(x_{1},\cdots ,x_{b}),$ and $l^{b}\left(x_{1},\cdots ,x_{b}\right)$ is the codeword length for the block $x_{1}^{b}.$

For simplicity, in this section, all the properties and algorithms will be given for a source code of a random variable. However, they are valid or trivially extended to the block source code for a random vector as in the Definition 2.

For the case D=2 (see Definition 1), l(x) records the number of bits used to represent C(x). Since *X* is a random variable, it is possible to determine the expected length of the code C. Moreover, it is expected to use the fewest number of elements to encode a message, it being necessary to adopt a criterion given by the next definition.

**Definition** **3.** *The expected length of the source code C for a random variable X with probability mass function p(x),x∈A, is given by*

$$E\left(l\left(X\right)\right)=\sum_{x\in A}p\left(x\right)l\left(x\right).$$

As expected, we want a code that provides a minimum expected length E(l(X)).

Necessarily, a *C* code that does not lead to ambiguity is one that verifies the following definition and such a code is called nonsingular.

**Definition** **4.** *A source code C, under the Definition 1, is a nonsingular code if, given $x,x^{\prime }\in A,$$x\neq x^{\prime }\Rightarrow C\left(x\right)\neq C\left(x^{\prime }\right).$*

Sending sequences of realizations of *X* would imply imposing certain conditions on a code that can be composed by the concatenation of codes of values of a random variable.

**Definition** **5.** *Given a source code under the Definition 1, and given a sequence of realizations of X, say $x_{1},x_{2},\ldots ,x_{n},$ the extension of C on the sequence is $C\left(x_{1}x_{2}\ldots x_{n}\right)=C\left(x_{1}\right)C\left(x_{2}\right)\ldots C\left(x_{n}\right),$ being the second term, the concatenation of the codes $C\left(x_{1}\right),C\left(x_{2}\right),\ldots ,C\left(x_{n}\right).$*

Under Definition 5, we set the expected conditions to consider as ideal a source code.

**Definition** **6.** *Under the Definition 1, the code C is said to be uniquely decodable if its extension, given by Definition 5, is nonsingular (see Definition 4).*

As we can see, Definition 6 can be ideal, but the constraint is that such a code is not necessarily instantaneous. Then, it can be necessary to read the whole codified message to proceed to decode it. This brings us to the notion of prefix (or instantaneous) code.

**Definition** **7.** *Under the Definition 1, the code C is said a prefix code if no codeword is a prefix of any other codeword.*

According to the various classifications attributed to source codes, the prefix codes are the strictest one (see [7]), in particular, it is a class that verifies the Definition 6. An example is the Huffman code, as shown in [7]. Furthermore, the prefix codes are related to Kraft’s inequality, via Theorem 5.2.1 (see [7]). A prefix code (under Definition 1) is such that $\sum_{x\in A}D^{-l(x)}\leq 1.$ That is, there is a conditioning on the possible values of l(x),x∈A. Thus, the Kraft’s inequality characterizes the prefix condition. Finding the ideal code (which must be prefix) is related to the minimization of

(1) $$\sum_{x\in A}p\left(x\right)l\left(x\right),$$

under the constraint

(2) $$\sum_{x\in A}D^{-l(x)}\leq 1\;\;\;\;\left(\mathrm{Kraf}t^{\prime }s\mathrm{inequality}\right),$$

then, the solution is given by $l\left(x\right)=-\log_{D}p\left(x\right),$ which implies in a possible non integer solution; thereby, the optimal size will be approximate. The first approximation is to consider the integer part of $\log_{D}p\left(x\right)$ for each codeword, say

(3) $$l^{*}\left(x\right)=-\left\lceil \log_{D}p\left(x\right)\right\rceil .$$

Consider the entropy of X,

(4) $$H_{D}\left(X\right)=-\sum_{x\in A}p\left(x\right)\log_{D}\left(p\left(x\right)\right).$$

If $E(l^{*}\left(X\right))$ is the expected length of a code following Equation (3), it verifies the Kraft’s inequality, guaranteeing $H_{D}\left(X\right)\leq E\left(l^{*}\left(X\right)\right)\leq H_{D}\left(X\right)+1,$ and, Theorem 5.2.1 (from [7]) ensures that there is an instantaneous code with these codeword lengths.

Any instantaneous code of expected length E(l(X)) satisfies $H_{D}\left(X\right)\leq E\left(l\left(X\right)\right),$ as demonstrated by Theorem 5.3.1 (from [7]), including the optimal code, with expected length $E(l^{opt}\left(X\right)),$ which in the set of instantaneous codes, verifies $E\left(l^{opt}\left(X\right)\right)\leq E\left(l\left(X\right)\right),$ for all E(l(X)) coming from an instantaneous code, while, by Theorem 5.4.1 (from [7]),

(5) $$H_{D}\left(X\right)\leq E\left(l^{opt}\left(X\right)\right)\leq H_{D}\left(X\right)+1,$$

where $E\left(l^{opt}\left(X\right)\right)=\sum_{x}l^{opt}\left(x\right)p\left(x\right).$

For a given distribution, an optimal prefix source code using the Huffman algorithm can be constructed, reproduced here (Algorithm 1), see also [8]. For simplicity, the algorithm is presented for a variable taking values from 1 to m, A={1,2,…,m} with D=2; then, the code is composed by concatenation of elements in {0,1}, those impositions can be easily eliminated in practice.
**Algorithm 1:** Huffman Code**Input**: P={p(1),p(2),…,p(m)},G={1,2,…,m} 1  If |P|=1,C(1):=0
 2  Otherwise, while |P|>1
   –   define
      ∗  I:=argmin{p(i)∈P:i∈G}
      ∗  J:=argmin{p(i)∈P∖{p(k):k∈I}:i∈G}
      ∗  H:=I∪J
   –   If |H|=2 set l(h):=0∀h∈H,C(I):=0 and C(J):=1
   –   Otherwise, if h∈I,C(h):=0C(h) and if h∈J,C(h)=1C(h).
   –   Set $I^{\prime }:=H,\;$$P^{\prime }:=\left\{p\left(k\right):k\in I^{\prime }\right\},\;$$p\left(I^{\prime }\right)=\sum_{k\in I^{\prime }}p\left(k\right),\;$$G^{\prime }=\left\{k:k\in I^{\prime }\right\}$
   –   Define $P:=\left\{P\setminus P^{\prime }\right\}\cup \left\{p\left(I^{\prime }\right)\right\},\;\;\;$$G:=\left\{G\setminus G^{\prime }\right\}\cup \left\{I^{\prime }\right\}$ and return to 1.
 **Output**: {C(1),C(2),…,C(m)}


Where |M| denotes the cardinal of the set M. The canonical Huffman code (CHC) is a modification of the Huffman code (Algorithm 1), such that the only information needed to decode it is the set of codelengths.

Consider, again, the random variable *X* taking values on the alphabet A={1,2,…,m}. Assume that the probabilities are not increasing such that, if i<j, then P(X=i)≥P(X=j). This assumption can be made without loss of generality because, if it is not true for X, there is a permutation π of A, such that Y=X∘π has no increasing probabilities.

Let {C(i),i∈A} be a Huffman code for X, with codelengths $l_{i}=l\left(C\left(i\right)\right),i=1\ldots m.$ Letting cal $C^{\prime },$ the corresponding CHC, $C^{\prime }$ is defined from ${\left\{l_{i}\right\}}_{i=1}^{m}$ in the following way:

(a) For $i=1,\;C^{\prime }\left(i\right)$ is the concatenation of $l_{1}$ zeroes.
(b) For j=i+1, assign $C^{\prime }\left(j\right)$ as the next (larger) binary number. Do this until the first k∈A such that the codelength increases, and append zeros to the right of $C^{\prime }\left(k\right)$ until $l\left(C^{\prime }\left(k\right)\right)=l_{k}.$
(c) repeat (b), until i=m.

For more information about the canonical Huffman code, see Section 5.8 in [7].

The following example illustrates the stages followed for the construction of the codes.

**Example** **1.** *Consider X a random variable with five values, listed in the alphabet A={a,b,c,d,e} with probabilities $p\left(a\right)=\frac{1}{11}=p\left(e\right),\;$$p\left(b\right)=\frac{4}{11},\;$$p\left(c\right)=\frac{3}{11},\;$$p\left(d\right)=\frac{2}{11}.$ We describe below the stages (i–iv) through which the coding process goes (using Algorithm 1).*

*i.*  *
$P=\{p\left(a\right)=\frac{1}{11},p\left(b\right)=\frac{4}{11},p\left(c\right)=\frac{3}{11},p\left(d\right)=\frac{2}{11},p\left(e\right)=\frac{1}{11}\},$ G={a,b,c,d,e}
*
  – *I=a,J=e,H={a,e}, and C(a)=0,C(e)=1*
  – *
$I^{\prime }=\left\{a,e\right\},p\left(I^{\prime }\right)=\frac{2}{11},G^{\prime }=\left\{a,e\right\}\Rightarrow$ $\left\{\begin{array}{c} P=\{p\left(I^{\prime }\right)=\frac{2}{11},p\left(b\right)=\frac{4}{11},p\left(c\right)=\frac{3}{11},p\left(d\right)=\frac{2}{11}\}\\ G=\{G^{\prime },b,c,d\} \end{array}\right.$
*
*ii*  *
$P=\{p\left(\left\{a,e\right\}\right)=\frac{2}{11},p\left(b\right)=\frac{4}{11},p\left(c\right)=\frac{3}{11},p\left(d\right)=\frac{2}{11}\},$ G={{a,e},b,c,d}
*
  – *I={a,e},J=d,H={{a,e},d}, and C(a)=00,C(e)=01,C(d)=1*
  – *
$I^{\prime }=\left\{a,e,d\right\},p\left(I^{\prime }\right)=\frac{4}{11},G^{\prime }=\left\{a,e,d\right\}\Rightarrow$ $\left\{\begin{array}{c} P=\{p\left(I^{\prime }\right)=\frac{4}{11},p\left(b\right)=\frac{4}{11},p\left(c\right)=\frac{3}{11}\}\\ G=\{G^{\prime },b,c\} \end{array}\right.$
*
*iii*  *
$P=\{p\left(\left\{a,e,d\right\}\right)=\frac{4}{11},p\left(b\right)=\frac{4}{11},p\left(c\right)=\frac{3}{11}\},$ G={{a,e,d},b,c}
*
  – *I=c,J={a,e,d},H={c,{a,e,d}}, and C(c)=0,C(a)=100,C(e)=101,C(d)=11*
  – *
$I^{\prime }=\left\{a,e,d,c\right\},p\left(I^{\prime }\right)=\frac{7}{11},G^{\prime }=\left\{a,e,d,c\right\}\Rightarrow$ $\left\{\begin{array}{c} P=\{p\left(I^{\prime }\right)=\frac{7}{11},p\left(b\right)=\frac{4}{11}\}\\ G=\{G^{\prime },b\} \end{array}\right.$
*
*iv*  *
$P=\{p\left(\left\{a,e,d,c\right\}\right)=\frac{7}{11},p\left(b\right)=\frac{4}{11}\},$ G={{a,e,d,c},b}
*
  – *
I=b,J={a,e,d,c}, H={b,{a,e,d,c}}.
*
*Obtaining the following Huffman code for X:*

C(b)=0,C(c)=10,C(a)=1100,C(e)=1101,C(d)=111.

*To find the canonical Huffman code, we need to re-code X (column 1 of Table 1) so that the probabilities are not increasing in lexicographic order, obtaining Y. The Huffman code for Y and the corresponding codelengths are obtained directly from C (see columns 5 and 6 of Table 1).*

*From the codelengths of C, we obtain the CHC, see column 7 of Table 1. Observe that the Huffman code and the canonical Huffman code share the same codelengths.*

The algorithm strategy is optimal, since, according to Theorem 5.8.1 (see [7]), if *C* is the Huffman code, or the CHC, and $\hat{C}$ another code that verifies the Definition 6 (uniquely decodable),

$$E\left(l^{C}\left(X\right)\right)\leq E\left(l^{\hat{C}}\left(X\right)\right),$$

where $l^{C}$ and $l^{\hat{C}}$ are the lengths of *C* and $\hat{C},$ respectively. That is, *C* offers the smallest expected size in the class of decodable codes. This means that elements less used in the encoding of the message occupy a greater number of bits than those used more by the message. It should be noted that optimality is not a guarantee of uniqueness, what Theorem 5.8.1 of [7] tells us is the first condition and not the second one.

In order to extend the idea to stochastic processes, we first introduce the appropriate notation and, after that, we adapt the coding process. Let $\left(X_{t}\right)$ be a discrete time order *o* Markov chain on a finite alphabet A, with o<∞.$S=A^{o}$ is the state space of the process $(X_{t}).$ Denote the string $a_{k}a_{k+1}\ldots a_{n}$ by $a_{k}^{n},$ where $\;a_{i}\in A,\;\;\forall i:\;k\leq i\leq n.$ In this context, the elements that define the process are the transition probabilities, for each a∈A and s∈S, define, $P\left(a|s\right)=Prob\left(X_{t}=a|X_{t-o}^{t-1}=s\right).$ Then, we have a set of probabilities to be identified, say

{P(a|s),a∈A,s∈S}.

According to this new context, it is necessary to implement the coding for each s∈S, as we show in the next example.

**Example** **2.** *Consider a process with memory o=1 and alphabet A={a,b,c}, with transition probabilities given by Table 2 (left):*

*Suppose that the message to be coded is ‘caabacba’. As the process has memory o=1, we do not have (transition) probabilities to use the canonical Huffman code on the first o elements. The first o elements are codified with the binary number corresponding to their lexicographic positions in the alphabet. In this case, o=1 and the first letter in the sequence is “c”, which corresponds to the third letter in the alphabet; as a result, the first element in the sequence has code 11 corresponding to 3 in binary.*

*The result is the string 111011101001110, which follows the strategy to read the string caabacba from left to right. c is coded by 11 (3 in binary), ca means that c is followed by a, then using Table 2a is coded by 10, aa means that a is followed by a, then a is coded by 11, etc. To decode the sequence 111011101001110, we need to consider the order o=1; then, the first element is decoded as c (third letter of the alphabet); now, we use the conditional line associated with c given in Table 2, so, if the symbol to decode has code 10 and previously we had c, then the original letter in the second position was a. In this stage, we use the conditional line associated with a; then, because the next symbol is 11, the letter with that conditional code is a, and so on.*

The example shows that the instantaneous coding is applicable to each conditional space of the $\left(X_{t}\right)$ process. That is, the process’ code is {C(x|s),s∈S,x∈A}, a collection of conditional codes. In addition, {l(x|s),s∈S,x∈A} is the collection of conditional lengths for the set of codes. That is, to identify the conditional codes C(a|a),C(b|a), and C(c|a), we consider the transition probabilities P(a|a)=1/6,P(b|a)=2/6 and P(c|a)=3/6 and, proceeding as in the previous example, we obtain C(a|a)=00,C(b|a)=01, and C(c|a)=1. Furthermore, this example shows how the initial part of the message that has not yet reached the minimum memory of the process can be treated. There are other ways to deal with the initial elements of a message, not necessarily like the one shown in the example, but such a question does not impact the contributions of the article. The procedure encodes one element at a time, with an optimal code depending on the past. It can easily be extended to *b* consecutive elements of the sample at a time, with an optimal Huffman code depending on the past. To calculate the conditional Huffman code for a block of *b* symbols, we first need to calculate the conditional transition probabilities for sequences of *b* letters of the alphabet.

Let $\left(X_{t}\right)$ be a discrete time order *o* Markov chain on a finite alphabet A, and state space $S=A^{o},$ with transition probabilities, {P(x|s),x∈A,s∈S}. Consider now $x_{1}^{b}\in A^{b},$ the transition probability $P(x_{1}^{b}|s),$ for the block $x_{1}^{b}$ given the state s∈S. This probability can be calculated from the set of transition probabilities {P(x|s),x∈A,s∈S}, in the following way:

$$P\left(x_{1}^{b}|s\right)=\prod_{i=1}^{b}P\left(x_{i}|{\left(sx_{1}^{b}\right)}_{i}^{i+o-1}\right),$$

where ${\left(sx_{1}^{b}\right)}_{k}^{j},k\leq j$ is the concatenation of the elements in positions k,k+1,⋯,j of the sequence resulting of the concatenation of $sx_{1}^{b}.$ This is done for each $x_{1}^{b}\in A^{b},s\in A^{o},$ to obtain $\{P\left(x_{1}^{b}|s\right),x_{1}^{b}\in A^{b}\}.$ After that, the set of transition probabilities for size *b* blocks is used to calculate $\left\{C^{b}\left(.|s\right),s\in A^{o}\right\}$, the conditional block Huffman code for the state *s* for each $s\in A^{o},$ applying the Huffman algorithm to the set of probabilities $\{P\left(x_{1}^{b}|s\right),x_{1}^{b}\in A^{b}\}.$

The Huffman code for blocks is relevant since Theorem 5.4.2 ([7]) claims that the expected code length per symbol, for a size *b* block Huffman code, converges to the entropy of the process, which is the theoretical minimal code length per symbol. For more information, see [9,10,11], and Section 5 in [7]. In our simulations, we will use blocks of size 4 to encode the data.

In the next section, we present the strategy for estimating transition probabilities, in a real message or data set. We also present the Partition Markov Model (PMM) that allows us to design the proposal for reducing the number of bits in the transmission of a message, using canonical Huffman encoding.

## 3. Stochastic Estimation Strategy and Optimal Coding

In practice, we need to estimate the transition probabilities; then, let $x_{1}^{n}$ be a sample of the process $\left(X_{t}\right),\;s\in S,$a∈A and n>o. We denote by $N_{n}\left(s,a\right)$ the number of occurrences of the state *s* followed by *a* in the sample $x_{1}^{n}$; this is $N_{n}\left(s,a\right)=|\left\{t:o<t\leq n,x_{t-o}^{t-1}=s,x_{t}=a\right\}|.$ In addition, the number of occurrences of *s* in the sample $x_{1}^{n}$ is denoted by $N_{n}\left(s\right)$ with $N_{n}\left(s\right)=|\left\{t:o<t\leq n,x_{t-o}^{t-1}=s\right\}|.$ Thus, for each a∈A and s∈S,$\frac{N_{n}\left(s,a\right)}{N_{n}\left(s\right)}$ is the estimator of P(a|s).

A reliable estimation of probabilities allows a precise coding, since the proposal of the Algorithm 1 depends on an adequate quantification of the probabilities, to develop the method of attribution of codes in an accurate way. In search of such precision, the idea of partitioning the state space is incorporated, associated with a stochastic process. The proposal aims to use different states, all elements of one specific part of that partition, to estimate the same probability. Let $L=\{L_{1},L_{2},\ldots ,L_{\left|L\right|}\}$ be a partition of S,∀a∈A and L∈L, define $P\left(L,a\right)=\sum_{s\in L}\mathrm{Prob}\left(X_{t-o}^{t-1}=s,X_{t}=a\right),\;$$P\left(L\right)=\sum_{s\in L}\mathrm{Prob}\left(X_{t-o}^{t-1}=s\right).$ If P(L)>0, we define $P\left(a|L\right)=\frac{P(L,a)}{P(L)}.$ Then, ∀a∈A,P(a|L)=P(a|s)∀s∈L, meaning that we use all the states s∈L to estimate the same parameter. However, which is the strategic partition? We formalize the notion in the next definition.

**Definition** **8.** *Let $\left(X_{t}\right)$ be a discrete time order o (o<∞) Markov chain on a finite alphabet A, and state space $S=A^{o},$*

*1.*  *s,r∈S are equivalent if P(a|s)=P(a|r)∀a∈A.*
*2.*  *$\left(X_{t}\right)$ is a Markov chain with partition $L=\{L_{1},L_{2},\ldots ,L_{\left|L\right|}\}$ if this partition is the one defined by the relationship introduced by item 1.*

This way to represent a stochastic process $\left(X_{t}\right)$ is called a Partition Markov Model.

Given Definition 8, it is clear that there will be a reduction in the number of probabilities necessary to describe the Markov chain in comparison with the full Markov chain that is to say that the cardinal |{P(a|L),a∈A,L∈L}| is smaller than or equal to the cardinal |{P(a|s),a∈A,s∈S}|. In order to obtain {P(a|L),a∈A,L∈L}, previously, it is necessary to identify L, which leads us to the estimation process that is detailed next. Once the structure L is identified, we can calculate the probabilities of the parts L:L∈L, using them in Algorithm 1, for encoding the message.

In a similar way to the one mentioned at the beginning of this section, the number of occurrences of elements in *L* followed by *a* and the total number of occurrences of elements in *L* are given by $N_{n}\left(L,a\right)=\sum_{s\in L}N_{n}\left(s,a\right),\;$$N_{n}\left(L\right)=\sum_{s\in L}N_{n}\left(s\right),\;\;L\in L.$ Then, the estimation of P(a|L) is given by $\frac{N_{n}\left(L,a\right)}{N_{n}\left(L\right)}$, which shows the benefit in terms of precision to calculate such probability.

The model given by Definition 8 is studied in [6]. The challenge is to find a way to produce a strong consistent estimation of L. In that paper, the proposal is to use the Bayesian Information Criterion (BIC), see [12], to achieve this goal. The BIC is formulated as follows:

(6) $$\begin{array}{c} \mathrm{BIC}\left(L,x_{1}^{n}\right)=\ln\left(\mathrm{ML}(L,x_{1}^{n})\right)-\frac{\left(|A|-1)|L\right|}{2}\ln\left(n\right), \end{array}$$

with $\mathrm{ML}\left(L,x_{1}^{n}\right)=\prod_{L\in L,a\in A}{\left(\frac{N_{n}\left(L,a\right)}{N_{n}\left(L\right)}\right)}^{N_{n}\left(L,a\right)},\;\;for\;\;N_{n}\left(L\right)\neq 0,\;L\in L.\;$$\mathrm{ML}(L,x_{1}^{n})$ is the pseudo maximum likelihood given a partition L with sample $x_{1}^{n}.$ The logarithm (ln) of the pseudo maximum likelihood term can also be written as

$$\ln\left(\prod_{L\in L,a\in A}{\left(\frac{N_{n}\left(L,a\right)}{N_{n}\left(L\right)}\right)}^{N_{n}\left(L,a\right)}\right)=\sum_{L\in L,a\in A}\ln\left({\left(\frac{N_{n}\left(L,a\right)}{N_{n}\left(L\right)}\right)}^{N_{n}\left(L,a\right)}\right).$$

It is shown in [6] that, by maximizing the BIC, it is possible to obtain a strong consistent estimate of the partition L. In practice, this maximization is attainable because of a characteristic of the BIC criterion that allows the definition of a BIC-based metric between candidate parts (see [6]). The metric is used in a clustering algorithm, like the one proposed in [5], to produce a consistent estimation of L. It is relevant to note that the BIC criterion allows the consistent estimation in other models of the area of inference in processes, see [13,14].

**Remark** **1.** *The Partition estimation problem studied in [6] is beyond the scope of this paper, but it is important to note that the estimation algorithms require to compute the frequency ratios,*

$$\frac{N_{n}\left(s,a\right)}{N_{n}\left(s\right)},\forall a\in A,\;s\in S.$$

*The number of frequency ratios (|A|−1)|S| should be much smaller than n to have confidence in the calculations. In practice, this restricts the maximal memory o admissible for the model used to compress the data, $o<\lfloor \log_{\left|A\right|}\left(n\right)\rfloor -1.$ In Section 5 (situation 6) with n=1000, we see an example of a model too large for the sample size.*

Now, and within the framework of stochastic processes, we establish the link between the length of a code and the entropy of the process, as established by Equation (5), for random variables.

The notion of entropy plays a fundamental role in the theory of codes and, as we will show below, it allows us to identify the expected codeword length per symbol of a message. For a stochastic process $(X_{t}),$ the entropy is given by

(7) $$\begin{array}{c} H_{D}\left(X_{t}\right)=\lim_{n\to \infty }\frac{H_{D}\left(X_{1},X_{2},\ldots ,X_{n}\right)}{n}. \end{array}$$

Using some basic properties of entropy, see [7], it is possible to prove the following result. The result allows for identifying the entropy of the process, under the partition structure given by Definition 8.

**Theorem** **1.** *Let $\left(X_{t}\right)$ be a discrete time order o (o<∞) Markov chain on a finite alphabet A, and state space $S=A^{o},$ with partition L following Definition 8. Then,*

$$H_{D}\left(X_{t}\right)=-\sum_{L\in L}P\left(L\right)\sum_{a\in A}P\left(a|L\right)\log_{D}\left(P\left(a|L\right)\right).$$

**Proof.**  By Theorem 4.2.1 in [7],

(8) $$\begin{array}{cc} \;\;\;\;\;\;\;\;\;\;\;\;\;\;\;\;\;H_{D}\left(X_{t}\right) & =\lim_{n\to \infty }H_{D}\left(X_{n}|X_{1},X_{2},\ldots ,X_{n-1}\right) \end{array}$$

(9) $$\begin{array}{cc} \; & \;\;\;\;\;\;\;\;\;\;\;\;\;\;\;\;\;\;\;\;\;\;\;\;\;\;\;\;\;\;\;\;\;\;\;=\lim_{n\to \infty }H_{D}\left(X_{n}|X_{n-o},X_{n-o+1},\ldots ,X_{n-1}\right) \end{array}$$

(10) $$\begin{array}{cc} \; & =H_{D}\left(X_{o+1}|X_{1}^{o}\right) \end{array}$$

(11) $$\begin{array}{cc} \; & \;\;\;\;\;\;\;\;\;\;\;\;\;\;\;\;\;\;\;\;\;\;\;\;\;\;\;\;\;\;\;\;\;\;\;=\sum_{s\in A^{o}}P\left(s\right)H_{D}\left(X_{o+1}|X_{1}^{o}=s\right)\\ \; & \;\;\;\;\;\;\;\;\;\;\;\;\;\;\;\;\;\;\;\;\;\;\;\;\;\;\;\;\;\;\;\;\;\;\;=-\sum_{s\in A^{o}}P\left(s\right)\sum_{a\in A}P\left(a|s\right)\log_{D}\left(P\left(a|s\right)\right)\\ \; & \;\;\;\;\;\;\;\;\;\;\;\;\;\;\;\;\;\;\;\;\;\;\;\;\;\;\;\;\;\;\;\;\;\;\;=-\sum_{L\in L}P\left(L\right)\sum_{a\in A}P\left(a|L\right)\log_{D}\left(P\left(a|L\right)\right) \end{array}$$

(8) follows from Theorem 4.2.1 in [7], (9) and (10) are consequence of the process having order *o* and for being stationary (see Equation (4.25) of [7]), (11) follows from the definition of conditional entropy. □

Under the assumptions of Theorem 1, and given a sample $x_{1}^{n}$ of the process $\left(X_{t}\right)$, we can estimate the entropy of the process by

(12) $$\begin{array}{c} {\hat{H}}_{D}\left(X_{t}\right)=-\frac{1}{n\times \ln(D)}\ln\left(\mathrm{ML}\left(\hat{L},x_{1}^{n}\right)\right), \end{array}$$

where $\hat{L}$ is the estimation of L.

Define $H_{L}=-\sum_{a\in A}P\left(a|L\right)\log_{D}\left(P\left(a|L\right)\right),L\in L,$ we obtain from Theorem 1

(13) $$H_{D}\left(X_{t}\right)=\sum_{L\in L}P\left(L\right)\times H_{L}.$$

Defining ${\hat{H}}_{L}=-\sum_{a\in A}\frac{N_{n}\left(L,a\right)}{N_{n}\left(L\right)}\log_{D}\left(\frac{N_{n}\left(L,a\right)}{N_{n}\left(L\right)}\right),L\in \hat{L},$ we build the plug-in estimator of $H_{D}\left(X_{t}\right)$

$$\begin{array}{cc} {\hat{H}}_{D}\left(X_{t}\right) & =\sum_{L\in \hat{L}}\frac{N_{n}\left(L\right)}{n}\times {\hat{H}}_{L}\\ \; & =-\frac{1}{n}\sum_{L\in \hat{L}}N_{n}\left(L\right)\sum_{a\in A}\frac{N_{n}\left(L,a\right)}{N_{n}\left(L\right)}\log_{D}\left(\frac{N_{n}\left(L,a\right)}{N_{n}\left(L\right)}\right)\\ \; & =-\frac{1}{n}\sum_{L\in \hat{L},a\in A}\log_{D}\left({\left(\frac{N_{n}\left(L,a\right)}{N_{n}\left(L\right)}\right)}^{N_{n}\left(L,a\right)}\right)\\ \; & =-\frac{1}{n\times \ln(D)}\sum_{L\in \hat{L},a\in A}\ln\left({\left(\frac{N_{n}\left(L,a\right)}{N_{n}\left(L\right)}\right)}^{N_{n}\left(L,a\right)}\right)\\ \; & =-\frac{1}{n\times \ln(D)}\ln\left(\prod_{L\in \hat{L},a\in A}{\left(\frac{N_{n}\left(L,a\right)}{N_{n}\left(L\right)}\right)}^{N_{n}\left(L,a\right)}\right)\\ \; & =-\frac{1}{n\times \ln(D)}\ln\left(\mathrm{ML}\left(\hat{L},x_{1}^{n}\right)\right). \end{array}$$

That is, the estimation of the partition $\hat{L}$ under the effect of $x_{1}^{n}$ allows us to identify an estimator of the entropy of the process, which leads us to ask if we can estimate an expected code size. For random variables, there is a relationship between $H_{D}$ and the expected length of the optimal code, see Equation (5). For such adaptation, consider an instantaneous block source code for the random vector $\left(X_{1},X_{2},\ldots ,X_{n}\right)$ (Definition 2), with codelength function l. Let $R_{n}$ be the expected codeword length per symbol,

(14) $$R_{n}=\frac{E(l\left(X_{1},X_{2},\ldots ,X_{n}\right))}{n}.$$

According to Theorem 5.4.2 ([7]), the minimum expected codeword length per symbol for an optimal block code $R_{n}^{opt},$ verifies $R_{n}^{opt}\to_{n\to \infty }H_{D}\left(X_{t}\right).$ Then, we obtain the next corollary.

**Corollary** **1.** *Under the assumptions of Theorem 1, the minimum expected codeword length per symbol $R_{n}^{opt}$ satisfies*

$$R_{n}^{opt}\to \sum_{L\in L}P\left(L\right)\sum_{a\in A}P\left(a|L\right)\log_{D}\left(\frac{1}{P(a|L)}\right),$$

*when n→∞, where $R_{n}^{opt}$ is given by Equation (14) and computed for an optimal block source code.*

**Remark** **2.** *If we consider the estimator of $H_{D}\left(X_{t}\right)$ given by Equation (12), $R_{n}^{opt}$ can be approximate by $-\frac{\ln(\mathrm{ML}\left(\hat{L},x_{1}^{n}\right))}{\ln(D^{n})},$ which is proportional to the first term of the BIC value of Equation (6), using the estimated partition $\hat{L},$ and with a negative sign.*

The compressed representation of the dataset will be composed of two parts—first, a description of the model used to code the data, and second the coded data. The minimum expected codeword length per symbol refers to the encoding of the data. Remark 2 shows that, for given data, the minimum expected codeword is smaller for models with larger values for the maximum likelihood.

**Remark** **3.** *A model with redundant parameters will require more bits for its description than the correct model. On the other hand, a model with redundant parameters usually has a maximum likelihood value larger than the correct model, and, because of Remark 2, will produce a shorter expected code length for the data than the correct model. We will see in Section 5 that using models with redundant parameters for compression produces larger files because the decrease in the size of the portion of the compressed output corresponding to the encoding of the data are smaller than the increase in the size of the part of the output with the specification of the model.*

The strategy that we investigate in this paper is supported by Definition 8 and the BIC criterion, whose tendency is to decrease the number of probabilities necessary for the encoding of a message compared to the use of a full Markov chain. While in the case of a full Markov chain such probabilities are (|A|−1)|S|, the same does not happen when approaching the problem from the perspective of a PMM, since the total number of probabilities depends on the cardinal of estimated L, representing a total equal to (|A|−1)|L|, and |L|≤|S|. In practice, if a full Markov chain is used, the encoding of the message occurs by Algorithm 1, provided as inputs $\{N_{n}\left(s,a\right),a\in A,s\in S\}.$ However, if a PMM is used, the encoding of the message is given by Algorithm 1, provided as inputs $\{N_{n}\left(L,a\right),a\in A,L\in \hat{L}\},$ where $\hat{L}$ is the estimate of L.

In the next section, the purpose is to describe the process of transmitting a message. The first subsection shows to the reader the steps that need to be considered. Later, we present the advantages of using the counts $\{N_{n}\left(L,a\right),a\in A,L\in \hat{L}\},$ related to a PMM instead of using the counts $\{N_{n}\left(s,a\right),a\in A,s\in S\},$ related to a full Markov process.

## 4. Codification of the Model Information

In this section, we show how we encode the model and parameters. For simplicity and clarity of exposition, we will assume that the receiver knows the alphabet A, and there is no need to encode *A* in the message to be transmitted. In the case of an order *o* Markov chain, the model is determined by the order o. In the case of a PMM, the model is determined by the partition $\hat{L}$ of the state space. In both cases, the parameters of the model are the estimated transition probabilities. In our case, as the transition probabilities are estimated from the message to encode, the parameters will be the transition frequencies $\left\{N_{n}\left(s,a\right),a\in A,s\in S\right\}$ in the case of an order *o* Markov chain, and $\left\{N_{n}\left(L,a\right),a\in A,L\in \hat{L}\right\}$ in the case of a PMM. Note that this is not a review of the ways to implement the coding/decoding process, since our goal is to show how we can incorporate and take advantage, in the compression process, of the notion of partition (Definition 8).

### 4.1. Codification of the Transition Frequencies

To send a coded sequence of numbers, we need a protocol so that the receiver knows where the code of each number starts and finishes. If we send the integer *k* in binary, the receiver has to know how many bits we are using to encode that number. For example, the integer 5 can be encoded as 101 or as 00101. For any integer k, the minimal number of bits needed to represent *k* in binary is equal to $\lfloor \log_{2}\left(k\right)\rfloor +1$ bits. We know that $N_{n}\left(s,a\right)\leq n,\forall \;a\in A,s\in S,$ and $N_{n}\left(L,a\right)\leq n,\forall \;a\in A,L\in \hat{L}$; this means that each frequency, on any possible model for the data, can be encoded in $\lfloor \log_{2}\left(n\right)\rfloor +1$ bits. In what follows, all the frequencies will be encoded with a fixed number $\left(\left\lfloor \log_{2}\left(n\right)\right\rfloor +1\right)$ of bits. For example, to send 30 frequencies, it will be used $30\times (\left\lfloor \log_{2}\left(n\right)\right\rfloor +1)$ bits.

### 4.2. Codification of the Model

In the case of an order *o* Markov chain model, the model is determined by the order o. We will assume that the order is smaller than $\log_{\left|A\right|}\left(n\right)-1$; this means that it can be written in $\lfloor \log_{2}\left(\log_{\left|A\right|}\left(n\right)-1\right)\rfloor +1$ bits or less. We will reserve $\lfloor \log_{2}\left(\log_{\left|A\right|}\left(n\right)-1\right)\rfloor +1$ bits for the order *o* of the model.

In the case of a PMM, to send the model, we need to send the partition, that is, the relationship between the states s∈S and the parts L∈L. In the next example, we show how to do that through the canonical Huffman code.

Now we detail our proposal. If $\hat{L}$ is the estimated partition for the state space S such that $\hat{L}=\left\{L_{1},L_{2},\ldots ,L_{|\hat{L}|}\right\}$, then we associate to each state s∈S an index i, if $s\in L_{i}.$ The states are arranged in lexical order, and this list is replaced by the indices previously assigned. As each $L_{i}$ has a cardinal, which is the total number of states contained in it, say $|L_{i}|,$ each *i* is associated with a probability $\frac{|L_{i}|}{\left|S\right|},$ and the canonical Huffman code is applied to these frequencies. Finally, the index list is replaced by the canonical Huffman code list and communicated by a string (say C(Index)) in the transmission process. Example 3 illustrates the process.

**Example** **3.** *Consider the process $\left(X_{t}\right)$ over the alphabet A={0,1}, state space $A^{o},$ with order o=3. Suppose that under the Definition 8 the partition $L=\{L_{1},L_{2},L_{3}\}$ is identified, with the composition given by Table 3.*

*Considering the lexical order of the elements, we associate to each state s the value i, if $s\in L_{i},$*

*Then, the list of indices is 12211231 (see Table 4), and each index can be coded by the canonical Huffman algorithm, since we have associated with each index a frequency. See the results reported in Table 5.*

*As a consequence, to indicate to which part each state belongs, we need to codify the sequence* 12211231 *with the codes in column 4 of Table 5. The string to communicate is given by (15),*

(15) $$\begin{array}{c} 0\;\;10\;\;10\;\;0\;\;0\;\;10\;\;11\;\;0, \end{array}$$

*which requires 12 bits. To inform the codification, we need to send the codelengths. In this case, we send the first codelength (1) and then the increments (last column in Table 5). The string is 110, which requires 3 bits. In total to inform the partition, we need to communicate 15 bits, plus $\lfloor \log_{2}\left(\log_{2}\left(n\right)-1\right)\rfloor +1$ bits for the order.*

The next example complements the previous one showing how using a PMM (see Definition 8 and Table 3) can cause a reduction in the number of bits necessary for the transmission of the model.

**Example** **4.** *Consider that the partition of Table 3 was generated from a size n=393 sample, with the frequencies represented by Table 6, as the information that generates the partition reported in Example 3.*

*First, suppose we want to transmit the model and parameters using an order 3 Markov chain. To communicate the model, we need to transmit the order using $\;3\;=\;\lfloor \log_{2}\left(\log_{2}\left(393\right)-1\right)\rfloor +1$ bits. In order to communicate the counts reported in Table 6, we use the strategy previously described. We use a fixed number of bits to represent each $N_{n}\left(s,a\right),a=0,1,s\in S.$ For example, with n=393, we can produce the binary representation of $N_{n}\left(s,a\right)$ with $\;9\;=\lfloor \log_{2}\left(393\right)\rfloor +1$ bits for each a=0,1 and s∈S. Then, the string that is communicated is built from the concatenation in lexical order of the representations of each count, fixing first the state and plugging all the representations following the lexical order of the elements of the alphabet:*

(16) $$\begin{array}{c} 000011110\;\;000010100\;\;000011000\;\;000011001\;\;000011010\;\;000011001\;\;000011111\;\;000010100\\ 000011101\;\;000010100\;\;000011001\;\;000011001\;\;00001010\;\;000011110\;\;000011110\;\;000010100 \end{array}$$

*Then, the string requires 16×9=144 bits. Adding the 3 bits for the order of the model, we get the total of 147 bits.*

*In conclusion, in the case of using an order 3 Markov chain model, the total number of bits required for the specification of the model plus the parameters is 147 bits.*

*Now, suppose that we want to transmit the model and parameters using a partition Markov model. Considering the partition structure of Table 3 plus the frequencies in Table 6, we have the frequencies in Table 7:*

*Then, under this new scheme that considers the counts related to the parts, this is $N\left(L,a\right)=\sum_{s\in L}N\left(s,a\right),$ the string that needs to be communicated is the concatenation of the binary representations of the six frequencies in Table 3, written using $9=\lfloor \log_{2}\left(393\right)\rfloor +1$ bits for each N(L,a),a=0,1 and L∈L, The strings is*

(17) $$\begin{array}{c} 001111000\;\;001010000\;\;001001011\;\;001001011\;\;000001010\;\;000011110. \end{array}$$

*The number of bits that the configuration needs is 6×9=54 bits.*

*From the concatenation of strings 011 corresponding to the order, plus the string (15) corresponding to the codification of the partition, and (17) corresponding to the frequencies, we obtain the final string, encoding the partition Markov model,*

011010100010110001111000001010000001001011001001011000001010000011110.

*In addition, the string requires 69 bits.*

*Table 8 summarizes the results under two perspectives, using a regular order 3 Markov chain versus the approach by partition:*

We see then, from the previous example, that the compression produced by the approach (Definition 8) allows for reducing some costs if there is a reasonable number of parts that allows for reducing the information to be transmitted, in comparison with the model with all the states separated, or with a shy reduction.

In the next section, we show simulation studies and an application illustrating the use of Huffman code allied to partition Markov models, and we describe its performance in each situation. We compare the performance of the Huffman coding under two strategies, that is, we compare the performance of the Huffman code when it is implemented using a full order *o* Markov chain versus using a partition Markov model.

## 5. Simulation Study and Application

### 5.1. Simulation Study

In the next simulations, we generate samples $x_{1}^{n},$ of sizes n=1000,5000, and 10,000, for each setting simulated—following some specific stochastic model, given for each case by a partition Markov model, since a PMM is a generalization of full Markov models.

We implement the two strategies for comparison. The first called *by States* is the Huffman code applied to a full order *o* Markov chain, executed on the transition probabilities estimated from the sample, state by state. The second is called *by Parts*, which is the Huffman code executed on the transition probabilities estimated from the sample, part by part, see Definition 8 and Equation (6).

In all the cases (we consider eight settings), the codification of the dataset was made using the Huffman code in blocks of size 4.

On the simulations, we want to check the influence of the number of parameters of the simulated model on the resulting compression. In the case of a full order *o* Markov chain over the alphabet A, the number of parameters is (|A|−1)|S|. In the case of a Partition Markov model, the number of parameters is (|A|−1)|L|. We simulate eight models with the following principles. The first two models have the same number of parameters for both families of models—meaning that each part contains a single state. This could be considered the worst condition for the *by Parts* methodology. On the contrary, the third and four models have tiny partitions with only two parts; these two models seem very favorable to the PMM as the models can be described with a minimal number of parameters. Even more favorable to the PMM is a small partition and large state space; scenarios implemented in cases 5 and 6, with state space of size 64 and 256, respectively, and partitions of size 4. Finally, in models 7 and 8, we consider the case of having a small state space and small partition. In that scenario, both strategies should work well.

For each sample size and case, we performed 100 independent simulations and applied the two strategies for the sample. In Table 9, we report the mean number of bits used for the compression of the model (columns 6 and 7), the mean number of bits used for the compression of the dataset (columns 8 and 9), and the mean compression ratio, defined as

$$R_{c}=\frac{\mathrm{uncompressed}\mathrm{size}}{\mathrm{compressed}\mathrm{size}}.$$

**Simulation 1.**  Situations 1 and 2, with Markov partitions L having the same size as the state space S.

*The two first situations we show in Table 9 could be considered at first, unfavorable to the partition structure since the number of parts is equal to the number of states. Nonetheless, as we can see, the rates are better for the *by Parts* methodology being that the BIC criterion chooses a model with a set of parameters that are considered relevant from the point of view of the likelihood and for a given sample size. Note how the number of bits reserved for the model grows with the size of the dataset; consider a fixed distribution, for larger datasets, the number of bits dedicated to the header can be larger. In addition, since the BIC criterion is consistent, there is a sample size such that, from that sample size on, the number of parameters will not increase, only the number of bits needed to transmit larger frequencies. In the two situations, we can observe a huge difference in the number of bits being used to describe the models, columns 6 and 7 in Table 9. Again, this is caused by the BIC criterion restricting the parameters to the ones that are relevant, for the likelihood of the sample. It produces the reverse effect on the size of the codified data.*

*We can also observe that, for situation 2, the number of parameters to be codified for the strategy *by States* is so large (256×4 transition frequencies) that it does not compensate the compression of the data $x_{1}^{n},$ producing a compression ratio smaller than 1 for all three sample sizes. For the strategy *by Parts*, this only happens for the smaller sample size n=1000.*

**Simulation 2.**  Situations 3 and 4, with a cardinal of the Markov partitions L being very small.

*The situations corresponding to the models labeled as 3 and 4 in Table 9 could be considered as favorable to the partition structure. Scenarios with smaller partitions could induce a small size on the description of the estimated model. Looking at columns 6 and 7 in Table 9, we can see that, indeed, the number of bits required to encode the description of the model is small compared to situations 1 and 2, and also that it changes very little with the size of the dataset. This happens because the model selection mechanism detects and incorporates into the estimated model all the relevant parameters even for the smaller sample size n=1000.*

*As seen in Remark 3, given a set of models for the dataset, the model with the larger number of parameters will have a larger maximum likelihood value and a smaller minimum expected codeword length per symbol. We note that, even with a significant difference in the number of parameters, the differences in the length of the code corresponding to the data are relatively small. The reason is that the extra parameters on the model by States are irrelevant as they correspond to transition probabilities already considered in other states. In addition, the estimated transition probabilities are more precise for the Partition Markov model as they are drawn from a large sample corresponding to each one of the estimated parts.*

**Simulation 3.**  *Situations 5 and 6. Small Markov partitions L, and large state space S.*

*In both situations, the size of the partition generating the models is small |L|=4. However, the sizes of the state spaces are large, for situation 5, |S|=64, for model 6 |S|=256. The cases produce the larger differences in rates of compression, in the whole simulation study.*

*We also notice that, in the case of model 6 and sample size 1000, both methods are ineffective for data compression, as the rate of compression is lower than 1. The reason is in Remark 1, where we state that, to fit a PMM model, the order *o* should satisfy $o<\lfloor \log_{\left|A\right|}\left(n\right)\rfloor -1\left(=3\right)$. In the case of the model for States, we have a similar problem, the number of transition probabilities to be computed from the data are 256×3=768, almost the size of the dataset, which is 1000. We can see that, for n=1000, the number of bits for the data (column 9) is disproportionately larger for the methodology *by Parts*, which shows that the BIC criterion could not find a good model for the sample. The situation pointed before is solved for sample sizes* 5000 *and 10,000.*

**Simulation 4.**  Situations 7 and 8. Small Markov partitions L, and small state space S.

*For both models, we use |S|=8. The rates of compression for both approaches are similar and show the efficiency of the compression procedure. For sample size 1000, the rates for the method *by Parts* are significantly better as the codification of the model is more significant for the smaller dataset. For larger sample sizes, there is a small advantage for the methodology *by Parts*, approaching the limit given by the entropy of the process. The difference, in favor of the methodology *by Parts*, is caused by the codification of the model as seen in columns 6 and 7 in Table 9. The difference in the size of the codification of the data is very small, see columns 8 and 9 in Table 9.*

Two facts can be seen through Table 9. The first one is that the number of bits needed for the transmission of the model is always smaller for the method *by Parts*. This happens because of the ability of the Bayesian Information Criterion to incorporate parameters into the model, according to its relevance to describe the dataset.

Second, the number of bits needed to transmit the data for the *by Parts* structure is always larger than for the *by States* structure. The cause is stated in Remark 2, which says that the negative maximum likelihood estimator $-\frac{\ln(ML\left(\hat{L},x_{1}^{n}\right))}{\ln(D^{n})}$ can approximate the optimal expected codeword length per symbol $R_{n}^{opt}.$ As the partition model has a number of parameters smaller than or equal to the standard order *o* Markov chain, the maximum log-likelihood is smaller or equal for the partition Markov model, which, through Remark 3, produces an expected code length larger or equal for the partition Markov model. This effect is compensated by the smaller size of the model provided by the conception of the partition Markov model, promoting a reduction in the number of bits needed by the approach *by Parts* in comparison to the traditional one *by States* (values in bold in Table 9).

### 5.2. SARS-CoV-2 and Compression

In this application, we use as a background the current health crisis produced by SARS-CoV-2, which affects the entire world. Sites like https://www.worldometers.info/coronavirus/ (accessed on 24 December 2021) record the enormous number of deaths and daily cases that humanity faces. The magnitude of this situation has led scientists to genetic analysis to identify the alterations that are leading today to genetic mutations of the virus, which potentiate its distribution capacity and its capacity to lead to death. Ref. [4] records that, for example, in Brazil, alterations in the genetic structure of SARS-CoV-2 were already detected at the first half of 2020. Today, such mutations are factors that jeopardize the efficacy of vaccines against SARS-CoV-2, developed during 2020. We begin 2021 with the uncertainty of the course that this epidemic may take. Locations in an uncontrolled situation can be controlled if ways are found to inhibit the evolution of new strains of the virus.

Counting on the rapid communications that occur today between scientists around the world and the volume of genetic information available, this application aims to contribute to the development of this type of analysis, considering genetic databases such as the one available in NCBI (National Center for Biotechnology Information, accessed on 24 December 2021), via link https://www.ncbi.nlm.nih.gov/.

**Figure 1:**
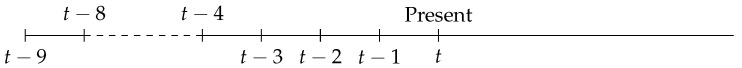
Scheme of the past necessary to determine the partition of the state space of the SARS-CoV-2 process at time t, in dashed line, the irrelevant period with limits on top of the scheme [t−8,t−4].

Specifically in this article, we use three genetic sequences, in the *Fasta* format—one from China and the other two collected in Brazil. Our selection is based on the discoveries made in [3,4] that have allowed us to know the stochastic behavior of these sequences. The sequence MN908947.3, coming from a patient of Central Hospital in Wuhan (Hubei, China), can be obtained from the free base NCBI. It is the first SARS-CoV-2 complete genome sequence registered in the literature, see [15]. In [3], we model this sequence (using its *Fasta* format), and the resulting model is a PMM model with interstice on the alphabet {a,c,g,t}. As introduced in [3], the model was selected using the BIC criterion. This model is very natural for genetic behavior. To the usual parameter *o* (see Definition 8), we add a new parameter G,G>o, which means that a piece of extra information about the behavior of the sample further in the past needs to be incorporated into the model to better catch the stochastic behavior of the sequence. In this case, o=3 (since we deal with the genomic alphabet, which is organized in triples), and G=9 (see table 6 (right) in [3]). Then, in the compression process, we need to report two parameters o=3 and G=9. We have a continuous past of size o=3 and a gap between positions 4 and 8 in the past, the value in the position G=9 being used to define the states of the state space, as illustrated in Figure 1. Each state of the state space is composed of a value (from the alphabet A={a,c,g,t}) associated with the distant past located 9 positions behind position *t* and a triple of values related to the positions t−3,t−2, and t−1; then, each state is $s\in A\times A^{3}.$

In [3], this model is widely investigated within the framework of the sequence MN908947.3 of SARS-CoV-2. It results in a model made up of 13 parts, where each part gathers states with the constitution described above. In the present application, we use the same model that was also addressed later in [4], for the other sequences investigated here, MT126808.1 (patient traveled from Switzerland and Italy (Milan) to Brazil) and MT350282.1 (also from a patient in Brazil).

The link http://www.ime.unicamp.br/~veronica/PMM_Covid19/ (accessed on 24 December 2021) exposes for each Brazilian sequence the content of the partitions and their transition probabilities. We see that all the sequences have approximately the same number of estimated parts; MN908947.3 and MT350282.1 have 13 parts and MT126808.1 has 12 parts.

In Table 10, we present a summary of the compression process. We do not report the bits for the alphabet, as it is the genetic alphabet {a,c,g,t} itself. For the memory parameters, *o* and G, we have allocated 4 and 8 bits, respectively, both for strategy *by States* and for strategy *by Parts*.

We observe that, for the three genetic sequences of SARS-CoV-2, there is a reduction in the total number of bits that exceeds 10.4%, when comparing the strategy *by States* with the strategy *by Parts*. The situation here is due to the fact that the number of bits destined to encode the model required for strategy *by Parts* is much lower than the number of bits destined to encode the model required for strategy *by States*.

One aspect that could be considered in the compression process of many sequences of the same virus is to use a model that determines the parts considering a generalization of the Definition 8, such as the notion introduced in [16]. The notion introduced in [16] proposes to determine the parts using any sequence and any state, joining in the same part sequences and states if they share the transition probabilities. With such a model in hand, the bits used for the description of the model would be informed only once, remaining only to inform the coding of the sequences.

## 6. Conclusions

In this paper, we propose an improvement in the Huffman coding system’s efficiency, taking into account that the method uses transition probabilities that are computed from a message to be transmitted. A message has a dependent structure for which the element *a*, that is, a letter of the alphabet, a∈A, which is in the position *t* that occurs depending on the past, *o* values of the message, $s\in A^{o}$ ($S=A^{o}$ the state space). The Huffman coding system uses the transition probabilities P(a|s) to compress the message without loss. To improve the Huffman coding system in a Markov chain setting, we introduce the notion of Partition (see Definition 8, Section 3), which is a parsimonious structure on the state space that allows using several states to identify the same probability P(a|L), where *L* contains several states. The proposal is to reduce the number of bits needed to transmit the frequencies that estimate the transition probabilities associated with the message. Section 4.2 shows and exemplifies the strategy.

On the other hand, there is a potential loss of efficiency due to needing to identify which part *L* each state *s* belongs to, and, depending on the context, the number of bits used for such transmission must be considered. These situations are investigated in Section 5. We conclude that the strategy, via Definition 8, compensates, since a model with redundant parameters will use more bits for the description of the model than the correct one. We report in Section 5 that, for all the situations studied, the partition strategy (*by Parts*) improves the compression concerning a full Markov chain *by States*. In addition, except for the two cases in which the sample size is similar to the number of transition probabilities, we need to estimate to choose a model, the strategy *by Parts* has a compression rate significantly larger than 1.

Using Theorem 1, we show that the entropy of a process can be expressed using the structure of a partition. By estimating such a model, we can estimate the entropy of a process, which is the generator of a message, see Equation (12). A partition Markov model is identified through the Bayesian Information Criterion (see Equation (6)), showing a clear relationship with the minimum expected codeword per symbol (see Corollary 1), which can be estimated using the Bayesian Information Criterion and the estimated partition $\hat{L},$ see Remark 2. These results allow us to estimate the minimum expected codeword per symbol through the estimation process of a partition Markov model.

We complete this article with a compression application in the framework of SARS-CoV-2 sequences (see Section 5.2). We see that the procedure proposed in this article is capable of producing a reduction of more than 10% in compression in relation to Huffman code without the incorporation of the partition Markov model notion. The strategy shows promise in times of need for extra agility in the transmission and modeling of genetic sequences, as is the case of this application.

As a legacy of this article, we can say that the development of new models, such as the Partition Markov model, as well as estimation methods with relevant properties, such as the Bayesian Information Criterion, provide enough substance to contribute in the field of data compression, and this article aims to show this fact.

## Figures and Tables

**Table 1:** Huffman and canonical Huffman for X. From left to right: x,P(X=x),*y*, P(Y=y), Huffman code for Y, codelength for Y, CHC for *Y*$\left(C_{Y}^{\prime }\right)$, and codelength increments.

*x*	P(X=x)	*y*	P(Y=y)	$C_{Y}\left(y\right)$ (Huffman)	l(y)	$C_{Y}^{\prime }\left(y\right)$ (CHC)	l(y) Increment
*b*	4/11	*a*	4/11	0	1	0	1
*c*	3/11	*b*	3/11	10	2	10	1
*d*	2/11	*c*	2/11	111	3	110	1
*a*	1/11	*d*	1/11	1100	4	1110	1
*e*	1/11	*e*	1/11	1101	4	1111	0

**Table 2:** Left: transition probabilities P(x|s). Right: conditional canonical Huffman code C(x|s) and length l(x|s),x∈A={a,b,c},s∈S={a,b,c}.

	P(a|s)	P(b|s)	P(c|s)	C(a|s)	l(a|s)	C(b|s)	l(b|s)	C(c|s)	l(c|s)
s=a	1/6	2/6	3/6	11	2	10	2	0	1
s=b	1/3	1/3	1/3	10	2	11	2	0	1
s=c	2/7	1/7	4/7	10	2	11	2	0	1

**Table 3:** List of parts of $(X_{t}).$ The second column reports the composition of the part (on the left) and the third column reports the cardinal of the part.

Part ($L_{i}$)	Composition of $L_{i}$	$|L_{i}|$
$L_{1}$	{000,011,100,111}	4
$L_{2}$	{001,010,101}	3
$L_{3}$	{110}	1

**Table 4:** List of states in lexical order and related index.

*s*	000	001	010	011	100	101	110	111
index	1	2	2	1	1	2	3	1

**Table 5:** List of indices that identify the states with the parts reported in Table 3. The second column reports the frequency of the index (on the left). The third column reports the Huffman code of the index. The last three columns report the CHC, the codelengths, and the increments in codelengths, respectively.

Index	Frequency	Huffman	CHC	Codelength	Increase in Codelength
1	4/8	1	0	1	1
2	3/8	01	10	2	1
3	1/8	00	11	2	0

**Table 6:** Counts (and binary form) of each state *s* followed by 0 (and 1), from a message $x_{1}^{n},$ also represented by partition in Table 3—in blue, the cases of part $L_{1},$ in red the cases of part $L_{2}$, and in magenta the case of part $L_{3}$.

s→	000	001	010	011	100	101	110	111
$N_{n}\left(s,0\right)$	30	24	26	31	29	25	10	30
binary	11110	11000	11010	11111	11101	11001	1010	11110
$N_{n}\left(s,1\right)$	20	25	25	20	20	25	30	20
binary	10100	11001	11001	10100	10100	11001	11110	10100

**Table 7:** Counts of each part *L* followed by 0 (and 1), from the message represented by the partition in Table 3.

L→	$L_{1}$	$L_{2}$	$L_{3}$
$N_{n}\left(L,0\right)$	120	75	10
binary	1111000	1001011	1010
$N_{n}\left(L,1\right)$	80	75	30
binary	1010000	1001011	11110

**Table 8:** $|\{N_{n}\left(s,a\right),s\in S,a\in A\}|,\;$$\left|\{N_{n}\left(L,a\right),L\in L,a\in A\}\right|$ and number of bits necessary for the transmission, for each case.

	$\left\{N_{n}\left(s,a\right),s\in S,a\in A\right\}$	$\left\{N_{n}\left(L,a\right),L\in L,a\in A\right\}$
Number of counts	16	6
Number of bits	147	69

**Table 9:** Results for the simulation study. From left to right. (1) Model description: label (situation), order, number of parts, and size of the alphabet. (2) Sample size (n) of $x_{1}^{n}.$ In the next columns, we report mean values over 100 samples. On the left for the strategy *by States*, on the right, for the strategy *by Parts*. (3) Bits for the model: cost in bits for the codification of the model (columns 6 and 7). (4) Bits for the data: cost in bits for the codification of the data $x_{1}^{n}$ (columns 8 and 9). (5) Bits (total): total of bits (columns 10 and 11). (6) Compression ratio $R_{c}$ (columns 12 and 13). In bold type, the cases with the smallest number of bits and higher $R_{c}$.

Model Description				*n*	Bits for the Model		Bits for the Data $x_{1}^{n}$		Bits (Total)		Compression Ratio ($R_{c}$)	
**Label**	o	|L|	|A|		**States**	**Parts**	**States**	**Parts**	**States**	**Parts**	**States**	**Parts**
				1000	2326.2	366.6	831.9	1121.5	3158.1	**1488.1**	0.64	**1.35**
1	3	64	4	5000	3143.5	829.2	4673.2	5131.6	7816.6	**5960.8**	1.28	**1.68**
				10,000	3401.5	1299.9	9380.3	9864.9	12,781.8	**11,164.8**	1.57	**1.80**
				1000	3415.9	567.3	653.7	1558.2	4069.6	**2125.5**	0.49	**0.94**
2	4	256	4	5000	11508.1	1069.4	4379.6	5842.1	15,887.7	**6911.5**	0.63	**1.45**
				10,000	13,449.8	1391.1	9206.2	11,103.7	22,656.0	**12,494.7**	0.88	**1.60**
				1000	713.0	116.7	1125.6	1166.1	1838.6	**1282.8**	0.82	**1.17**
3	3	2	3	5000	991.5	131.6	5777.3	5816.8	6768.8	**5948.4**	1.11	**1.26**
				10,000	1074.7	138.7	11,572.6	11,620.8	12,647.3	**11,759.5**	1.19	**1.28**
				1000	739.9	125.0	1127.9	1188.1	1867.8	**1313.1**	0.80	**1.14**
4	3	2	3	5000	989.3	131.5	5768.4	5807.9	6757.8	**5939.4**	1.11	**1.26**
				10,000	1076.3	137.7	11,598.4	11,645.5	12,674.6	**11,783.2**	1.18	**1.27**
				1000	2460.1	358.4	899.4	1060.7	3359.5	**1419.1**	0.60	**1.41**
5	3	4	4	5000	3144.4	395.6	5016.0	5168.5	8160.5	**5564.1**	1.23	**1.80**
				10,000	3400.5	411.6	10,173.0	10,341.4	13,573.5	**10,753.0**	1.47	**1.86**
				1000	3341.8	613.2	634.1	1842.6	3975.9	**2455.8**	0.50	**0.81**
6	4	4	4	5000	11,874.7	971.6	4531.5	5201.9	16,406.2	**6173.5**	0.61	**1.62**
				10,000	13,484.8	987.6	9709.9	10,371.0	23,194.8	**11,358.6**	0.86	**1.76**
				1000	158.6	99.5	549.9	555.7	708.5	**655.2**	1.41	**1.53**
7	3	6	2	5000	196.6	139.9	2737.0	2744.2	2933.6	**2884.1**	1.70	**1.73**
				10,000	212.6	176.0	5465.6	5469.9	5678.2	**5646.0**	1.76	**1.77**
				1000	157.8	74.3	564.8	570.2	722.6	**644.5**	1.39	**1.56**
8	3	3	2	5000	196.6	100.3	2804.1	2807.4	3000.7	**2907.6**	1.67	**1.72**
				10,000	212.6	106.9	5618.7	5625.5	5831.3	**5732.5**	1.72	**1.74**

**Table 10:** From top to bottom: sequence ($x_{1}^{n}$) name, sample size *n* of $x_{1}^{n},$ country of origin. On the left, for the strategy *by States*, on the right, for the strategy *by Parts*, (1) cost in bits for the codification of the model, (2) cost in bits for the codification of the data $x_{1}^{n},$ (3) bits (total)—in bold type, the cases with the smallest number of bits.

Sequence ($x_{1}^{n}$)	MN908947.3		MT126808.1		MT350282.1	
*n*	29,903		29,876		29,903	
Origin	China		Brazil		Brazil	
Strategy	States	Parts	States	Parts	States	Parts
Bits of Model	9168	1811	9169	1711	9169	1799
Bits of $x_{1}^{n}$	58,116	58,401	58,063	58,501	58,123	58,489
Bits (total)	67,284	**60,214**	67,232	**60,212**	67,292	**60,288**

## Data Availability

The National Center for Biotechnology Information Advances Science and Health, https://www.ncbi.nlm.nih.gov/ (accessed on 24 December 2021). https://www.worldometers.info/coronavirus/ (accessed on 24 December 2021). http://www.ime.unicamp.br/~veronica/PMM_Covid19/ (accessed on 24 December 2021).
